# Patient:innensicherheit – Entwicklung und Evaluation

**DOI:** 10.1007/s00103-025-04168-5

**Published:** 2025-12-03

**Authors:** Max Geraedts, Ruth Hecker, Cordula Mühr

**Affiliations:** 1 Institut für Gesundheitsversorgungsforschung und Klinische Epidemiologie, Fachbereich Medizin, Philipps-Universität Marburg, Karl-von-Frisch-Straße 4, 35043 Marburg, Deutschland; 2 Zentralbereich Qualitäts- und klinisches Risikomanagement, Universitätsklinikum Essen (AöR), Essen, Deutschland; 3 Patientenvertretung im Unterausschuss Qualitätssicherung des G-BA, Berlin, Deutschland

**Keywords:** Patient:innensicherheit, Versorgungsqualität, Unerwünschte Ereignisse, Patient:innenerfahrungen, CIRS, Patient safety, Healthcare quality, Adverse events, Patient experiences, CIRS

## Abstract

Unter Patient:innensicherheit (PaSi) wird heute nicht mehr nur die Vermeidung unerwünschter Ereignisse, sondern vielmehr alle Aktivität zur Gewährleistung einer sicheren Versorgungsumgebung und einer PaSi-Kultur verstanden. PaSi gilt als eines der vordringlichen Ziele der Gesundheitsversorgung. Folgerichtig bemüht sich eine Vielzahl von Akteuren der gemeinsamen Selbstverwaltung in Deutschland heute um die PaSi. Dabei reichen die Methoden zur Bestimmung der PaSi von Patient:innenbefragungen über freiwillige Meldungen der Leistungserbringenden mithilfe von Critical Incident Reporting Systems (CIRS) bis hin zu Analysen von Krankenakten und Routinedaten, während sich die Suche nach den Voraussetzungen einer sicheren Versorgung klassischer Methoden des Qualitätsmanagements bedient. Evaluationsergebnisse aus Deutschland deuten darauf, dass die meisten PaSi-relevanten Ereignisse (Patient Safety Incidents, PSI) unentdeckt bleiben und weiterhin ein großer Forschungsbedarf besteht, um PSI-Inzidenzen, Determinanten von PSI und Interventionen zur Vermeidung von PSI sowie Schaffung einer PaSi-Kultur zu untersuchen.

## Abstract (en)

Patient safety (PaSi) is no longer understood as merely the prevention of adverse events, but rather as all activities aimed at ensuring a safe care environment and a culture of patient safety. PaSi is considered one of the most pressing goals in healthcare. Consequently, a large number of stakeholders in joint self-administration in Germany are now working to improve PaSi. The methods used to determine PaSi range from patient surveys and voluntary reports by healthcare providers using critical incident reporting systems (CIRS) to analyses of medical records and routine data, while the search for the prerequisites for safe care makes use of classic quality management methods. Evaluation results from Germany indicate that most PaSi incidents (PSI) remain undetected and that there is still a great need for research to investigate PSI incidences, determinants of PSI, and interventions to prevent PSI as well as to create a PaSi culture.

## Einleitung

Aktuelle erschütternde Fälle, wie der Palliativarzt, der mindestens 15 Patient:innen tötete und dann zum Teil deren Wohnungen zur Vertuschung seiner Taten in Brand setzte, oder der Krankenpfleger, der mindestens 85 Patient:innen auf Intensivstationen tötete, weil er seine Reanimationsfähigkeit immer wieder erproben wollte, sind sicherlich Extrembeispiele für Gefährdungen der Patient:innensicherheit (PaSi). Dagegen machen die Schätzungen zur Epidemiologie von Fehlern in der Gesundheitsversorgung deutlich, dass das Thema PaSi jeden angeht. Aufhorchen ließ in den letzten Jahren die im *British Medical Journal* erschienene Schätzung, dass medizinische Fehler mit rund 250.000 Fällen pro Jahr die dritthäufigste Todesursache in den USA seien [1]. Die Autoren revidierten damit den oftmals als Ausgangsunkt der internationalen Aufmerksamkeit für das Problem der PaSi benannten Bericht des Institute of Medicine aus dem Jahr 1999 „To err is human: building a safer health system“ [2]. Dieser Bericht veranschlagte die Zahl der vermeidbaren Todesfälle durch medizinische Fehler in den USA noch mit unter 100.000 Fällen pro Jahr.

Für Deutschland gibt es noch immer keine Studie, die verlässliche Zahlen zu fehlerbedingten, vermeidbaren Todesfällen in der gesamten Gesundheitsversorgung berichtet. Es wird jedoch geschätzt, dass die für den Sachverständigenrat zusammengestellte Literaturanalyse, die für den Krankenhausbereich davon ausgeht, dass eine:r von Tausend Krankenhauspatient:innen vermeidbar zu Tode kommt [3], auch auf Basis neuerer Studien weiterhin zutrifft. Demnach sind jährlich etwa 17.000 fehlerbedingte Todesfälle allein im Krankenhaus zu erwarten [4].

Die Patient:innensicherheit muss also als eine zentrale Herausforderung der Gesundheitsversorgung und eines ihrer vordringlichen Ziele angesehen werden, ein Anliegen, das sowohl in Deutschland als auch international betont wird [5]. In diesem Beitrag wird die Entwicklung des Themas bis hin zu einer allgemeinen Übereinkunft beleuchtet. Zunächst wird das im Laufe der Jahre veränderte Konzept der PaSi erläutert, anschließend werden die wesentlichen Akteure der PaSi und Methoden zur Bestimmung der PaSi vorgestellt und empirische Befunde zur PaSi in Deutschland kursorisch dargestellt.

## Entwicklung des Konzepts Patient:innensicherheit

Die Sicherheit der Gesundheitsversorgung gilt schon seit Langem als eines der herausragenden Ziele von Gesundheitssystemen, als Dimension der Qualität der Versorgung, als zu prüfendes Outcome im Rahmen des Qualitätsmanagements der Gesundheitsversorgung und als eine der Dimensionen der Patient:innenzentrierung [6, 7]. Dem lag zunächst die knappe, inzwischen veraltete Definition von Patient:innensicherheit (PaSi) zugrunde, die diese als „Abwesenheit unerwünschter Ereignisse“ beschrieb – oder etwas ausführlicher gemäß der Definition der Weltgesundheitsorganisation (WHO) als „the reduction of risk of unnecessary harm associated with healthcare to an acceptable minimum“ [8]. Hierbei stand die Entdeckung von Fehlern im Vordergrund, die mithilfe von Methoden des Qualitäts- und Risikomanagements reduziert werden können.

Heute wird dagegen ein systemisches Konzept der PaSi vorgezogen, bei dem PaSi definiert wird als „ein Rahmen organisierter Aktivitäten, die Kulturen, Prozesse, Verfahren, Verhaltensweisen, Technologien und Umgebungen in der Gesundheitsversorgung schaffen, welche beständig und nachhaltig Risiken senken, das Auftreten vermeidbarer Schäden reduzieren, Fehler unwahrscheinlicher machen und die Auswirkungen von eintretenden Schäden verringern“ [5]. PaSi basiert letztlich auf einer (Patient:innen‑)Sicherheitskultur, worunter der WHO Global Patient Safety Action Plan Folgendes versteht: „Die Sicherheitskultur einer Organisation ist das Produkt aus individuellen und gruppenspezifischen Werten, Einstellungen, Wahrnehmungen, Kompetenzen und Verhaltensmustern, die die Merkmale des Gesundheits- und Sicherheitsmanagements der Organisation bestimmen. Organisationen mit einer positiven Sicherheitskultur zeichnen sich durch eine auf gegenseitigem Vertrauen basierende Kommunikation, durch eine gemeinsame Wahrnehmung der Bedeutung von Sicherheit und durch Vertrauen in die Wirksamkeit von Präventionsmaßnahmen aus“ [5].

Diese Definitionen legen bereits einen stärkeren Fokus auf die organisatorischen Voraussetzungen für eine sichere Versorgung, wie sie in den letzten Jahren zunehmend im Rahmen des Safety-II-Konzepts betont werden [9]. Die Initiatoren dieses Konzepts bezeichnen den traditionellen PaSi-Ansatz als „Safety-I“, bei dem es darum geht, Fehler zu erkennen, deren Ursachen und Determinanten zu bestimmen und daraus zu lernen, um in Zukunft Fehler zu verhindern. Dagegen betrachtet „Safety-II“ in der Medizin, warum unter den in der Gesundheitsversorgung gegebenen Bedingungen hoher Variabilität (der Patient:innen, der Leistungserbringenden, des gesamten Kontexts der Versorgung), Komplexität und daher ständiger Anpassungsvorgänge trotzdem so oft eine problemlose Versorgung gelingt. Die Faktoren, die zum Gelingen sicherer Versorgung beitragen, werden also fokussiert. Aus der Perspektive von Public Health könnte man davon sprechen, dass das Konzept der Patient:innensicherheit bei Safety-II dem salutogenetischen Ansatz von Antonovsky folgt, während bei Safety‑I eher der pathogenetische Ansatz vorliegt. Genauso könnte man aus der Perspektive des Qualitätsmanagements anführen, dass bei Safety-II die „Befähiger“ im Vordergrund stehen, wie sie beim Modell der European Foundation for Quality Management (EFQM; https://efqm.org) schon seit mehr als 35 Jahren benannt werden.

## Akteure der Patient:innensicherheit in Deutschland

Die genannten Konzepte gehen vor allem auf internationale Akteure wie die WHO oder die Organisation für wirtschaftliche Zusammenarbeit und Entwicklung (OECD) zurück. Aber auch in Deutschland ist eine Reihe von Akteuren im Bereich Patient:innensicherheit involviert, von denen hier einige exemplarisch genannt werden sollen.

Als aktueller Hauptakteur der PaSi in Deutschland muss sicher das Aktionsbündnis Patientensicherheit e. V. (APS) genannt werden. Das 2005 gegründete APS vereint eine Vielzahl Vertretender der Gesundheitsberufe, ihrer Verbände, der Patient:innenorganisationen sowie der Industrie und Wirtschaft. Interdisziplinär und multiprofessionell durchgeführte Projekte liefern die Basis für Fachinformationen sowie für Handlungsempfehlungen und Informationsbroschüren für Patient:innen und Leistungserbringende. Durch Kampagnen wie „Deutschland erkennt Sepsis“, „Aktion Saubere Hände“, „Speak up!“ oder „Stimmen für Patientensicherheit“ genauso wie die Veranstaltungen zum jährlichen Welttag der Patientensicherheit am 17. September trägt das APS maßgeblich zur öffentlichen Aufmerksamkeit zum Thema Patient:innensicherheit bei.

Von Beginn an waren Vertreter:innen der Patient:innenorganisationen im APS dabei, die ansonsten im Rahmen der Selbsthilfe Betroffenen beim Umgang mit patient:innensicherheitsrelevanten Ereignissen (Patient Safety Incidents, PSI) helfen oder seit 2004 durch ihre Mitwirkung in den Unterausschüssen (UA) des Gemeinsamen Bundesausschusses (G-BA), vor allem im UA Qualitätssicherung, eine patient:innenzentrierte Verfolgung des Ziels Patient:innensicherheit gewährleisten.

Als weiterer Verbundakteur, der mehr als 70 Organisationen im Gesundheitswesen, einschließlich Bundes- und Landesministerien sowie kommunale Verbände, vereint, ist das Forum „Gesundheitsziele.de“ zu nennen, dass im Jahr 2022 das Ziel „Patientensicherheit“ neu aufgenommen und dazu 2 Hauptziele (Patientensicherheitskultur und Patientensicherheitskompetenz) mit jeweils 6 Unterzielen definiert hat [10]. Die auf dem bereits genannten WHO Global Patient Safety Action Plan aufbauenden Ziele wurden aber noch nicht in Richtlinien des G‑BA aufgenommen und sind also bislang nur unverbindlich.

Die genannten Akteure engagieren sich zum Teil als Mitglieder der Dachorganisationen APS oder des Forums gesundheitsziele.de, zum Teil aber auch eigenständig. Hierunter sind das Bundesministerium für Gesundheit und die jeweils für Gesundheit zuständigen Landesministerien genauso wie die Partner der Selbstverwaltung im Gesundheitswesen zu nennen. Unter den gesetzlichen und privaten Krankenversicherungen hat beispielsweise die Techniker Krankenkasse (TK) zwischen 2019 und 2024 den „TK-Monitor Patientensicherheit“ herausgegeben, der jeweils auf der Basis einer repräsentativen Bürgerbefragung ausgewählte Aspekte zum Stand der Patient:innensicherheit erhoben hat [11, 12]. Der Verband der Ersatzkassen (vdek) bietet Patient:innen seit Februar 2024 in Zusammenarbeit mit der Deutschen Gesellschaft für Patientensicherheit im Rahmen eines befristeten Projekts ein Portal zur anonymen Rückmeldung von als kritisch erlebten Ereignissen an, das, einem Bericht in der *Ärztezeitung* vom 04.07.2025 folgend, nach Angaben des vdek im ersten Jahr wöchentlich 4 bis 12 Meldungen verzeichnete. Ein interprofessionelles Expert:innenteam bereitet aus den Rückmeldungen einzelne Fälle oder Fallgruppen auf und leitet daraus Verbesserungsmaßnahmen ab, die in Form von „Tipps für Versicherte“ oder als „Fall des Monats“ veröffentlicht werden (https://mehr-patientensicherheit.de/). Darüber hinaus unterstützen alle Versicherungen Patient:innen beim Verdacht auf einen Behandlungsfehler, wobei sie zur Begutachtung den Medizinischen Dienst beauftragen. Genauso kann man sich zur Abklärung von Behandlungsfehlervorwürfen an die Gutachter- und Schlichtungsstellen für ärztliche Behandlungen bei den Landesärztekammern wenden. Die Bundesärztekammer betreibt mit CIRSmedical.de (Critical Incident Reporting System) das bundesweite einrichtungsübergreifende Berichts- und Lernsystem der deutschen Ärzteschaft für kritische Ereignisse in der Medizin, das als Netzwerk auch andere CIRS einzelner Bundesländer oder Fachgesellschaften einbindet (wie CIRS-berlin, CIRS-nrw oder CIRS-ains der Anästhesisten). Im Vorfeld von Begutachtungen steht auch die Stiftung Unabhängige Patientenberatung Deutschland (UPD) als Beratungsorgan zur Verfügung, bei der Patient:innen u. a. die möglichen Zugangswege zu solchen Abklärungen erfragen können.

Als oberstes Selbstverwaltungsorgan muss der G‑BA selber als Akteur der PaSi erwähnt werden. So bestimmt der G‑BA in Form von Richtlinien über die von allen Leistungserbringenden durchzuführenden Maßnahmen zur Aufrechterhaltung der PaSi, fördert über den Innovationsausschuss Forschung zur PaSi und beauftragt einen weiteren wesentlichen Akteur, das Institut für Qualitätssicherung und Transparenz im Gesundheitswesen (IQTIG), das unter anderem die verpflichtenden Maßnahmen zur Qualitätsmessung entwickelt und anhand von Indikatoren die Qualität und Sicherheit der Gesundheitsversorgung in ausgewählten Leistungsbereichen darstellen soll.

Das IQTIG arbeitet in seinen Expert:innengruppen eng mit den medizinischen Fachgesellschaften und Vertretenden weiterer Gesundheitsberufe zusammen, die wiederum zum Teil selber Akteure der PaSi sind, indem sie in ihren Leitlinien das Thema PaSi berücksichtigen, Register zur Beurteilung der PaSi betreiben oder aber im Rahmen der Initiative „Gemeinsam Klug Entscheiden“ solche Prozeduren benennen, die mit Gefährdungen der PaSi in Form von Über- oder Unterversorgungen einhergehen können [13]. Beispielhaft für eine Fachgesellschaft mit einem besonderen Schwerpunkt im Bereich der PaSi sei die Gesellschaft für Qualitätsmanagement im Gesundheitswesen (GQMG) genannt, die sich mit Arbeitshilfen, Positionspapieren, dem „GQMG-Assessment-Tool zum klinischen Risikomanagement“®, verschiedenen Fortbildungsformaten und ihrem Qualitätsmanagement-Curriculum für das Medizinstudium aktiv um die Steigerung der PaSi und PaSi-Kompetenz bemüht [14]. Zur Verankerung des Themas Patient:innensicherheit und Risikomanagement im Nationalen Kompetenzbasierten Lernzielkatalog Medizin (NKLM) hat gleichzeitig das Aktionsbündnis Patientensicherheit einen wesentlichen Beitrag geleistet.

Nicht zuletzt müssen auch die Patient:innen selbst als Akteure der PaSi genannt werden. Nur sie können durch Rückmeldungen zu ihren Versorgungserfahrungen Aufschluss über PSI geben, die oftmals erst außerhalb der aktuellen Behandlungssituation offensichtlich werden [15]. Gerade auch die PSI an den Nahtstellen zwischen Versorgungssettings und -sektoren sowie der Schweregrad und die Folgen von PSI können nur mithilfe der Patient:innen als Akteure der PaSi offengelegt werden. Unter einem präventiven Gesichtspunkt können Kampagnen wie die zum „Speak up!“ von großer Bedeutung sein, wie sie z. B. 2024 vom APS angestoßen wurde. Patient:innen werden dadurch ermuntert, bei Unsicherheiten nachzufragen, gegebenenfalls eine zweite Meinung einzuholen, auf Hygienemaßnahmen bei sich und anderen zu achten und sich selbst aktiv an der Entscheidungsfindung ihrer Therapie zu beteiligen. Die aktive Entscheidungsbeteiligung von Patient:innen im Sinne des „Shared Decision Making“ (SDM) findet in ihrer präventiven Bedeutung für die Patient:innensicherheit derzeit noch wenig Beachtung, obwohl davon ausgegangen wird, dass durch eine gemeinsame Therapieentscheidung die Patient:innenkompetenz und Therapieadhärenz gesteigert werden können. Die systematische Implementierung von SDM im Versorgungsalltag könnte die patient:innenzentrierte Indikationsstellung befördern und dadurch maßgeblich dazu beitragen, Über‑, Unter- und Fehlversorgung zu vermeiden. Auch die Erfahrungen derjenigen, bei denen die Behandlung trotz der Komplexität der Versorgung fehlerfrei verlief, sind im Sinne des Safety-II-Konzepts hochrelevant. Daher wird eine strukturierte Erfassung der Patient:innenerfahrungen gefordert [16] und die Analyse der Voraussetzungen für die stärkere Einbindung von Patient:innen als Akteure der PaSi steht im Fokus aktueller Forschung [17, 18].

## Methodische Ansätze zur Bestimmung der Patient:innensicherheit

Einer der Wege, über die Patient:innen zu Akteuren der PaSi werden, besteht darin, deren Rückmeldungen über die Behandlung und eventuelle PSI zu erfragen. Zwar existiert bisher in Deutschland noch keine strukturierte, regelmäßige, alle Versorgungsbereiche umfassende Befragung zu Patient:innenerfahrungen, jedoch sind einzelne Umfragen und Entwicklungsprojekte exemplarisch zu nennen, die in Zukunft in einer solchen Befragung münden könnten.

Eine der repräsentativen Umfragen, der bereits erwähnte TK-Monitor Patientensicherheit (TK-Monitor), hat jeweils Grundfragen zur Häufigkeit, zum Sicherheitsgefühl und einzelnen aktuellen Themen gestellt. Für eine andere repräsentative Umfrage, die im Rahmen eines vom Innovationsausschuss geförderten Projekts stattfand, dessen Ergebnisse im folgenden Abschnitt berichtet werden, wurde ein detailliertes Befragungsinstrument für den ambulanten Sektor entwickelt [19]. Etwa zeitgleich hatte das Bundesministerium für Gesundheit die Entwicklung eines weiteren Befragungsinstruments für den ambulanten Sektor gefördert [20]. Der „Frag-mich“-Fragebogen ist frei verfügbar und kann von Arztpraxen genutzt werden. Eine weitere Entwicklung eines Instruments zur Erfassung von Patient:innenerfahrungen entstand kürzlich im Rahmen des OECD-Projekts „PaRIS“ (Patient-Reported Indicators Surveys), das ebenfalls den ambulanten Sektor und hier speziell chronisch Kranke ≥ 45 Jahre fokussiert und unter vielen anderen Fragen auch nach der Häufigkeit erlebter PSI fragt [21].

All diesen Entwicklungen gemeinsam ist, dass sie die Perspektive der Patient:innen auf die PaSi für unverzichtbar halten. Dem dennoch bei der Befragung von Patient:innen oftmals vorgebrachten Vorwurf, dass Patient:innen gar nicht in der Lage seien festzustellen, ob ein PSI bzw. ein Behandlungsfehler vorliegt, kann mit dem einfachen Argument des in der Soziologie oft zitierten Thomas-Theorems begegnet werden („If men define situations as real, they are real in their consequences“ [22]). Wenn also Patient:innen ein unerwünschtes Ereignis erleben und dies als unerwünschtes Ereignis oder gar als Behandlungsfehler empfinden, werden sie entsprechend reagieren, indem sie beispielsweise die Ärztin wechseln. Die Konsequenz aus der Situationsdefinition ist auf jeden Fall real und für das Gesundheitswesen aus einer patient:innenzentrierten Perspektive relevant.

Die systematische Erfassung von Patient:innenerfahrungen kann als eine relativ neue Methode zur PSI-Bestimmung betrachtet werden. Dagegen gilt der erstmals in der Harvard Medical Practice Study genutzte Ansatz, Krankenakten kriterienbasiert im Hinblick auf (vermeidbare) unerwünschte Ereignisse (V-/UE) zu untersuchen, als etablierte Methodik [23]. Auf eine zufällige Ziehung einer großen Stichprobe von Akten folgt eine erste Sichtung durch zumeist speziell geschulte Pflegekräfte, die die Akten anhand einer Liste von 18 möglichen Hinweisen auf ein V‑/UE durchsehen. Diese Hinweise werden von 2 Ärzt:innen unabhängig begutachtet und zugleich wird die Vermeidbarkeit eingeschätzt. Ein ähnliches strukturiertes Verfahren wird beim Global Trigger Tool (GTT) angewandt [24]. Auch hier gibt es eine 2‑stufige Beurteilung, wobei aber nur V‑/UE auf der Basis von durchgeführten Prozeduren, nicht solche durch Unterlassung einbezogen werden. Zudem unterscheiden sich der Begutachtungsprozess (erste Sichtung durch 2 Gutachter:innen, zweite Sichtung durch ein Team), die Anzahl Kriterien (hier 54) und die Stichprobe (eher klein, z. B. 20 Akten pro Monat).

Krankenakten können als eine Form versorgungsnaher Daten (VeDa) betrachtet werden, deren Analyse erlaubt, V‑/UE zu erkennen. Inzwischen wurden auch Versuche unternommen, die Trigger automatisch in elektronischen Krankenakten zu erkennen, wobei jedoch noch große Ergebnisunterschiede vorherrschten [25]. Mithilfe neuer KI-Methoden, besserer Krankenhausinformationssysteme und der nun durchgängig eingeführten elektronischen Patientenakten kann erwartet werden, dass sich diese Probleme in Zukunft überwinden lassen. Eine große Hilfe dabei wäre, dass eine einheitliche datengestützte Dokumentation eingeführt und diese auch geschult wird [26].

Eine andere Form von VeDa stellen Routinedaten dar, die für die Leistungsabrechnung an die Kostenträger gesandt werden. Ungeplante Wiederaufnahmen, Todesfälle oder sehr lange Krankenhausaufenthalte nach kleineren Routineeingriffen lassen sich leicht als Hinweise auf mögliche V‑/UE erkennen. Als Sekundärdaten, deren primärer Zweck die Vergütung ist, müssen die Daten aber immer vor diesem Hintergrund interpretiert werden.

In eine ähnliche Rubrik können die Daten aus speziellen Krankheitsregistern, aber auch die Daten aus der externen Qualitätssicherung (eQS) eingeordnet werden. Die von den Leistungserbringenden selbstberichteten Daten der eQS werden größtenteils dazu genutzt, PSI zu erkennen – allein 61 % der vom IQTIG mit Stand März 2025 für die datengestützte einrichtungsübergreifende Qualitätssicherung gemäß DeQS-Richtlinie benannten 472 Qualitätsindikatoren fokussieren die Patient:innensicherheit, indem sie vor allem dokumentierte schwerwiegende Komplikationen und Sterblichkeit messen. Wie alle selbstberichteten Daten sollten aber auch diese nur vorsichtig interpretiert werden – es handelt sich um Indikatoren, die kein direktes Maß der PaSi wiedergeben, sondern nur Hinweise auf die wahrscheinliche Kompromittierung von PaSi liefern. Noch zu wenig werden Strukturen und Prozesse erhoben und einrichtungsbezogen veröffentlicht, die zur Vermeidung von PSI beitragen und so Patient:innen und Angehörigen relevante Informationen für ihre Leistungserbringerauswahl zur Verfügung stellen könnten. Nach wie vor fehlen beispielsweise aussagekräftige Informationen zum Stand der Hygienequalität in Krankenhäusern. Der G‑BA war im Zuge der Änderung des Infektionsschutzgesetzes schon 2011 aufgefordert worden, dazu Indikatoren zu entwickeln und diese Informationen bereitzustellen.

Ebenfalls als Hinweise auf einen möglichen „Eisberg“, von dem nur die Spitze sichtbar wird, dienen die Critical Incident Reporting Systems (CIRS) als Methode zur Bestimmung der PaSi. Leistungserbringende oder auch die Patient:innen können diese Systeme (z. B. CIRSmedical.de, mehr-patientensicherheit.de) nutzen, um V‑/UE zu berichten, und diese für Lernzwecke zur Verfügung stellen. Beispiele für Erkenntnisse zu und aus CIRS werden im Abschnitt zur Evaluation benannt.

Zuletzt soll noch die teilnehmende und nichtteilnehmende Beobachtung als Möglichkeit zur Bestimmung von PaSi erwähnt werden, die jedoch selten zum Einsatz kommt. Dem steht der bekannte Hawthorne-Effekt entgegen, der besagt, dass Beobachtete typischerweise ihr Verhalten ändern, sodass beispielsweise die PaSi gefährdenden Verhaltensweisen wie eine mangelhafte Händehygiene unter einer teilnehmenden Beobachtung eventuell nicht zu erkennen sind.

## Evaluation – empirische Befunde zur Patient:innensicherheit in Deutschland

Auf der Basis einiger der beschriebenen Ansätze zur Bestimmung der PaSi liegen empirische Befunde vor, die aber insgesamt nur ein unvollständiges Bild darüber vermitteln, wie es um die PaSi in Deutschland steht. Im Folgenden werden exemplarisch einige Befunde auf der Basis einer selektiven Literaturrecherche dargestellt.

Die zuletzt genannten CIRS sind nicht darauf ausgelegt, die Epidemiologie der PaSi zu erfassen. Stattdessen sollen einzelne Berichte dazu dienen, dass andere aus den Vorkommnissen lernen. Dieser Lernzweck wird durch die Aufbereitung der Berichte in CIRSmedical oder aber auch im CIRS der Allgemeinmedizin „Jeder Fehler zählt“ erfüllt, wie Kowalski et al. in ihrer 17-Jahres-Analyse zeigen. Die Autoren weisen aber auch darauf hin, dass über den gesamten Zeitraum nur sehr wenige, nämlich nur 781 Berichte eingegangen waren [27]. Dagegen herrschen in der Anästhesie laut einer Umfrage von Marcus et al. eine fortgeschrittenere Fehlerkultur und Nutzung von CIRS vor. 54 % der befragten Anästhesist:innen gaben an, bereits mindestens einmal eine CIRS-Meldung abgegeben zu haben. Bemängelt wird jedoch von vielen Befragten, dass nach der Eingabe eines Ereignisses im Durchschnitt nur knapp 30 % der Meldungen eine Rückmeldung bzw. ein daraus resultierendes Lernen zur Folge haben [28].

Eine systematische Aufbereitung aller aus dem Bereich der PaSi erfassten Indikatoren der externen Qualitätssicherung liegt bisher nicht vor. Zwar werden die Ergebnisse aller (PaSi‑)Indikatoren aus den erfassten 9 Versorgungsbereichen mit insgesamt 16 QS-Verfahren gemäß der DeQS-Richtlinie vom IQTIG in den jährlichen Bundesauswertungen berichtet, jedoch werden die Daten nicht genutzt, um einen Überblick zum Stand der PaSi in diesen Versorgungsbereichen zu geben, die immerhin rund 20 % aller Krankenhausfälle in Deutschland umfassen [29]. Im groben Überblick wurden im Erfassungsjahr 2023 über alle Verfahren hinweg rund 8 % rechnerisch auffällige Indikatorergebnisse und davon nach den sogenannten Stellungnahmeverfahren etwa 19 % qualitativ auffällige Ergebnisse konstatiert, die oftmals die PaSi betrafen. Zudem existieren zumindest für einzelne Bereiche der Versorgung Hinweise, dass die externe Qualitätssicherung, insbesondere die Einführung der verpflichtenden Veröffentlichung der Indikatorergebnisse pro Klinik, in einzelnen Bereichen der Versorgung positive Effekte auf die Qualität und Sicherheit der Versorgung hat [30–32]. Diese Studien ergänzen die Übersichtsarbeit von Bauer und Gronemeyer, die die Ergebnisse von 13 Projekten aus Deutschland zusammenfasst, die quantitative Verbesserungen in verschiedenen Aspekten der PaSi erzielt haben [33]. Die Autoren konstatieren, dass es Interventionen gibt, die mit Verbesserungen der PaSi assoziiert sind, die Studien seien aber größtenteils von unzureichender Qualität, sodass bessere Studien- und Evaluationsdesigns sowie mehr Forschung nötig seien.

Im Gegensatz zu den IQTIG-Daten werden die Daten der Gutachterkommissionen und Schlichtungsstellen der Ärztekammern sowie die Daten der medizinischen Dienste systematisch aufbereitet. Diese letzteren Begutachtungen betreffen Behandlungsfehlervorwürfe, die von Patient:innen gegenüber den gesetzlichen Krankenkassen vorgebracht werden. Die Ergebnisse spiegeln wider, dass hier tatsächlich nur die Spitze des PaSi-Eisbergs zu erkennen ist. So erfassten die Schlichtungsstellen beispielsweise für das Jahr 2023 insgesamt nur 7529 Begutachtungsanträge, bei denen letztlich 1162-mal ein Behandlungsfehler und in 948 Fällen eine Kausalität bejaht wurde [34]. In einer etwas höheren, aber dennoch geringen Größenordnung befindet sich die Statistik der medizinischen Dienste. Im Jahr 2023 wurden von den Diensten 12.438 Behandlungsfehlervorwürfe begutachtet, bei denen in 3595 Fällen ein Fehler, in 3160 Fällen ein Schaden und in 2679 eine Kausalität anerkannt wurden [35].

Ein Drittel der vom Medizinischen Dienst und ein Viertel der von den Schlichtungsstellen begutachteten Behandlungsfehlervorwürfe betrafen 2023 den ambulanten Sektor. Aus diesem Sektor liegen verschiedene Befragungsstudien vor, die eine vollkommen andere Häufigkeit patient:innensicherheitsrelevanter Ereignisse (PSI), Schäden und Fehler in Deutschland vermuten lassen. Die bisher größte Studie befragte 10.037 Bürger ≥ 40 Jahre telefonisch nach erlebten PSI, Schäden und deren Folgen [36]. 14,2 % der Befragten hatten allein im letzten Jahr insgesamt 2589 PSI erlebt, die in 75 % mit einem Schaden einhergegangen waren und in 10 % zu einem Krankenhausaufenthalt geführt hatten. Rechnet man die Ergebnisse dieser bevölkerungsrepräsentativen Erhebung hoch auf die Population der ≥ 40-Jährigen in Deutschland, muss allein in dieser rund 47 Mio. Bürger umfassenden Bevölkerungsgruppe (56 % der Gesamtbevölkerung) mit 12,2 Mio. PSI im ambulanten Sektor und 1,2 Mio. ambulanten PSI-assoziierten Krankenhausaufenthalten pro Jahr gerechnet werden – eine Dimension, die die begutachteten Vorwürfe nicht erahnen lassen.

Die Ergebnisse dieser Studie werden durch die jährlich zwischen 2019 und 2024 durchgeführten, ebenfalls bevölkerungsrepräsentativen telefonischen Befragungen im Rahmen des TK-Monitors Patientensicherheit gestützt. Demnach befürchtet über die Jahre hinweg relativ konstant jeweils rund ein Drittel der Befragten, im ambulanten oder stationären Sektor einen Schaden aufgrund einer Behandlung zu erleiden [37]. In den Jahren 2020–2022 berichteten 24–32 % der Befragten konkret, in den letzten 10 Jahren einen (vermutlichen) Behandlungsfehler erlebt zu haben [38, 39].

Eine niedrigere Häufigkeit vermuteter Behandlungsfehler wurde in der Umfrage mit dem „Frag-mich“-Instrument ermittelt. Nur 2,9 % der zwischen Februar und Juni 2020 Befragten gaben an, dass bei ihnen in den letzten 12 Monaten ein Fehler bei der Behandlung aufgetreten war. Die Autoren erklären den Unterschied zu anderen Studien u. a. mit der COVID-19-Pandemie und der schriftlichen Befragung ohne Erläuterungen [40].

Die Reihe exemplarischer Studienbeispiele zur PaSi in Deutschland beenden 2 Studien zu Aspekten der Patient:innensicherheitskultur, die als „Befähiger“ zur PaSi aufgefasst wird. Einen klaren Zusammenhang zwischen Aspekten der PaSi-Kultur und den Outcomes der Patient:innen konnten Sturm et al. feststellen [41]. Jedoch machten Gambashidze et al. darauf aufmerksam, dass bei solchen Untersuchungen immer bedacht werden muss, welche Funktion in einer Gesundheitsorganisation die Befragungsteilnehmenden einnehmen, da diese mit der Wahrnehmung der PaSi-Kultur korreliert ist [42]. Wie immer bei Befragungsstudien, sollten also methodische Aspekte detailliert berichtet werden, damit das Risiko von Fehlinterpretationen reduziert wird.

## Zukünftige Verbesserung der Patient:innensicherheit

Um in Zukunft die Reichweite der benannten Initiativen sowie deren Wirksamkeit und Nachhaltigkeit im Sinne einer verbesserten Patient:innensicherheit zu erhöhen, ist eine Reihe von Anstrengungen auf den Weg zu bringen, von denen einige hier stichwortartig angeführt werden sollen. Vordringlich wäre zunächst eine systematische Erfassung von Patient:innenerfahrungen einzuführen, die explizit auch die Erfahrung mit sicherheitsrelevanten Ereignissen umfassen muss. Diese sollte Teil einer Indikatoren-basierten PaSi-Erfassung sein. Ein entsprechendes Kernindikatorenset wird derzeit im Rahmen des vom Innovationsausschuss beim G‑BA geförderten Projekts „PSI-BUND“ entwickelt.^1^ Dabei ist ein patient:innenzentriertes Vorgehen anzustreben, indem die Stimme der Patient:innen bereits bei der Gestaltung der Erfassung, aber insbesondere auch bei der Publikation der Ergebnisse und deren Interpretation einbezogen wird.

Damit die Ergebnisse Wirkung entfalten, sollte die Patient:innensicherheit in der Aus‑, Weiter- und Fortbildung aller Gesundheitsberufe eine Rolle spielen – hierbei sollten nicht nur deren Determinanten und Vermeidung thematisiert werden, sondern es sollten auch die Kommunikation mit betroffenen Patient:innen, betreuenden Angehörigen und Kolleg:innen sowie herausfordernde Gespräche geübt werden. Die Umsetzung von Aktivitäten zur Verbesserung der PaSi setzt in den Gesundheitseinrichtungen voraus, dass sich die Führungskräfte ihrer bedeutenden Rolle für die PaSi-Kultur in den Einrichtungen bewusst sind und diese aktiv fördern. Daneben bedarf es auf der politischen Ebene der Einsicht, dass Organisationen, die Aktivitäten zu Verbesserungen von Qualität und Patient:innensicherheit voranbringen, gefördert und insbesondere auch mit den notwendigen Mitteln ausgestattet werden müssen.

Nicht zuletzt müssen die gesundheitspolitischen Rahmenbedingungen auch im Kontext von Reformgesetzgebungen so gestaltet sein, dass Qualität und deren Sicherung im Interesse der Patient:innensicherheit im Fokus stehen und nicht hintenangestellt werden. Im Zuge der Reform des Patientenrechtegesetzes wäre es beispielsweise ein sinnvoller Schritt in Richtung einer verstärkten Patient:innenzentrierung der Bemühungen um Patient:innensicherheit, wenn der vom Patientenbeauftragten der Bundesregierung, Stefan Schwartze, angestoßenen Diskussion zur Senkung des Beweismaßes bei Behandlungsfehlervorwürfen von der sogenannten „richterlichen Überzeugung“ auf die „überwiegende Wahrscheinlichkeit“ eine Umsetzung folgen würde.

## Fazit

Patient:innensicherheit (PaSi) wird heute nicht mehr nur als Abwesenheit unerwünschter Ereignisse, sondern vielmehr als ein Konzept betrachtet, bei dem es um die Vermeidung PaSi-relevanter Ereignisse (PSI) durch Lernen aus fehlerfreien Abläufen geht, was wiederum die Etablierung einer PaSi-Kultur voraussetzt. Eine Vielzahl an Akteuren kümmert sich um die PaSi in Deutschland und setzt dabei unterschiedliche Erfassungsmethoden von Befragungen über freiwillige Meldungen (CIRS) bis hin zu Auswertungen von Routinedaten ein. Evaluationsergebnisse deuten darauf, dass die meisten PSI unentdeckt bleiben und weiterhin ein großer Forschungsbedarf besteht, um PSI-Inzidenzen, Determinanten der PSI und Interventionen zur Vermeidung von PSI zu untersuchen und damit auch die Patient:innenzentrierung der Gesundheitsversorgung zu stärken.

## Footnotes

1 https://www.aps-ev.de/aktivitaeten/projekte/psi-bund-bundesweites-patientensicherheitsindikatoren-kern-set/. Zugegriffen: 24.09.2025.
